# First person – Josh Currie

**DOI:** 10.1242/bio.046292

**Published:** 2019-07-15

## Abstract

First Person is a series of interviews with the first authors of a selection of papers published in Biology Open, helping early-career researchers promote themselves alongside their papers. Josh Currie is first author on ‘The *Prrx1* limb enhancer marks an adult subpopulation of injury-responsive dermal fibroblasts', published in BiO. This work was initially started in Elly Tanaka's lab but was completed in the lab of Tatiana Sandoval-Guzmán at the Center for Regenerative Therapies Dresden. Josh worked in Tatiana's lab in Dresden on the later portions of this project. He is now an assistant professor at Department of Cell and Systems Biology/Department of Biochemistry, University of Toronto, Canada, investigating how molecules and cells are coordinated in time and space to recreate and repair tissues after injury.


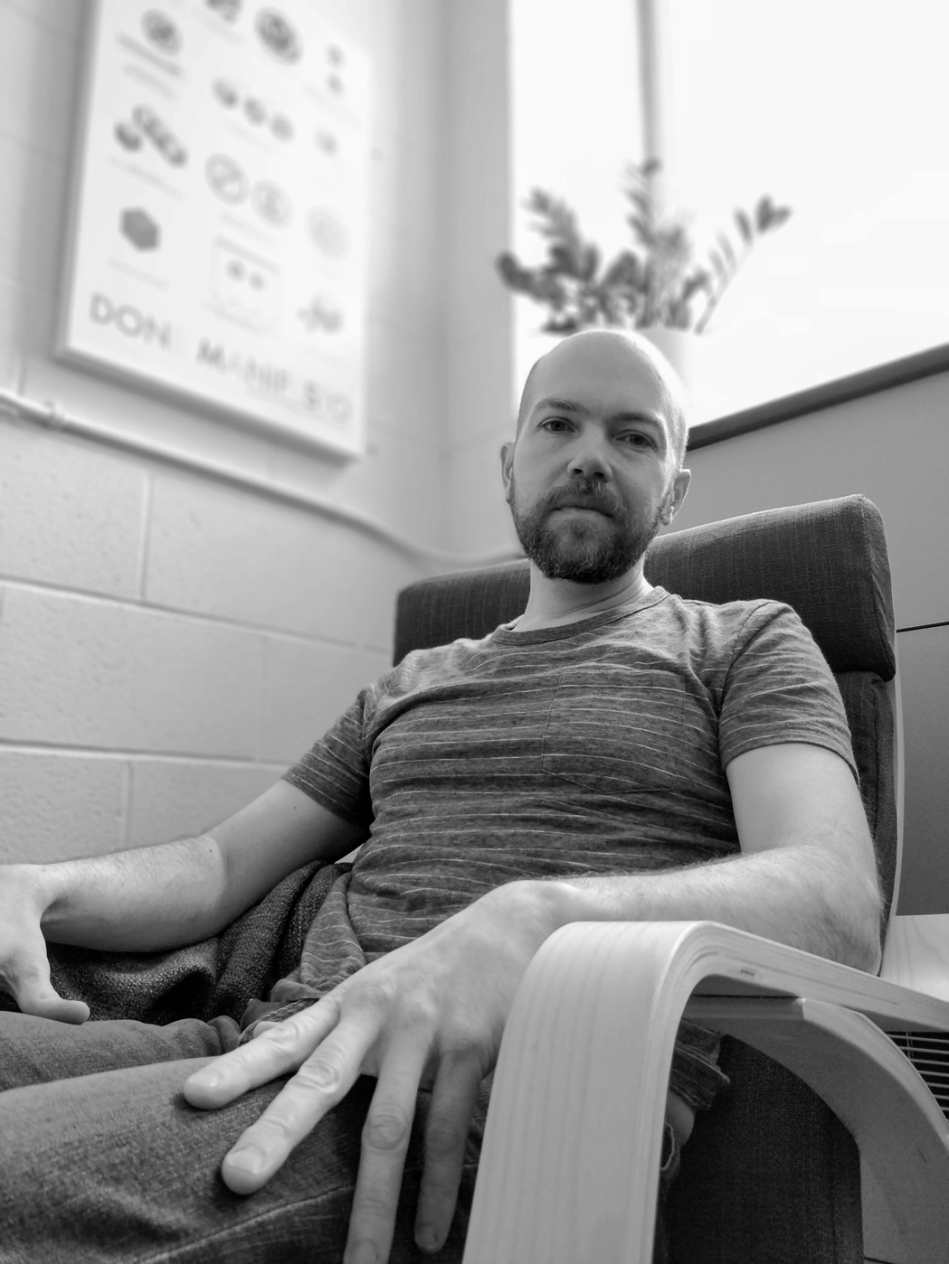


**Josh Currie**

**What is your scientific background and the general focus of your lab?**

I consider myself a card-carrying cell biologist and microscopist. I started out studying the actin and microtubule cytoskeleton as a graduate student at the University of North Carolina. For my postdoc, I decided that I wanted to understand how cells work together to pull off what I call ‘extreme acts of morphogenesis’. Complete limb regeneration certainly fits the bill! Josh worked in the lab of Elly Tanaka, then at the Center for Regenerative Therapies Dresden, working on both axolotl regeneration and mouse wound healing. In 2018, I started my lab at the University of Toronto to continue that work.

**How would you explain the main findings of your paper to non-scientific family and friends?**

Even a simple-looking tissue, like our skin, can be made up of incredibly diverse cells. We found a small population of cells in the mouse limb skin that retains a marker that was active during embryonic development. We found that these cells are responsive to injury and amplify within the wound far beyond the numbers and locations that they inhabited in the unwounded skin.

“Even a simple-looking tissue, like our skin, can be made up of incredibly diverse cells.”

**What are the potential implications of these results for your field of research?**

We describe a cell population that might be described as having MSC-like properties (mesenchymal stem cell) and they seem to have a positive contribution to wound healing. Hopefully, future studies will determine the functional role of these cells in wound repair and what additional molecular attributes set them apart from other cells.

**What, in your opinion, are some of the greatest achievements in your field and how has this influenced your research?**

There have been some heroic lineage-tracing studies of fibroblast populations in the last several years from Yuval Rinkevich, Ryan Driskell, Fiona Watt and Fabio Rossi, to name just a few. They all specifically explored cell function in an *in vivo* context, which is a difficult yet critical undertaking, and they have contributed key insights into the complex interactions that heterogenous cell types exhibit.

“There have been some heroic lineage-tracing studies of fibroblast populations in the last several years from Yuval Rinkevich, Ryan Driskell, Fiona Watt and Fabio Rossi…”

**What changes do you think could improve the professional lives of early-career scientists?**

I think that at graduate and postdoctoral levels, having more distributed mentoring is extremely beneficial for trainees. It can help resolve conflicts, keep projects on track and build support networks that can last your entire career. For a new group leader such as myself, I often wish that I had more administrative support. There's an overwhelming amount of administrative tasks, paperwork and bookkeeping that must be accomplished in addition to your true goal of getting your research moving.

**Figure:**
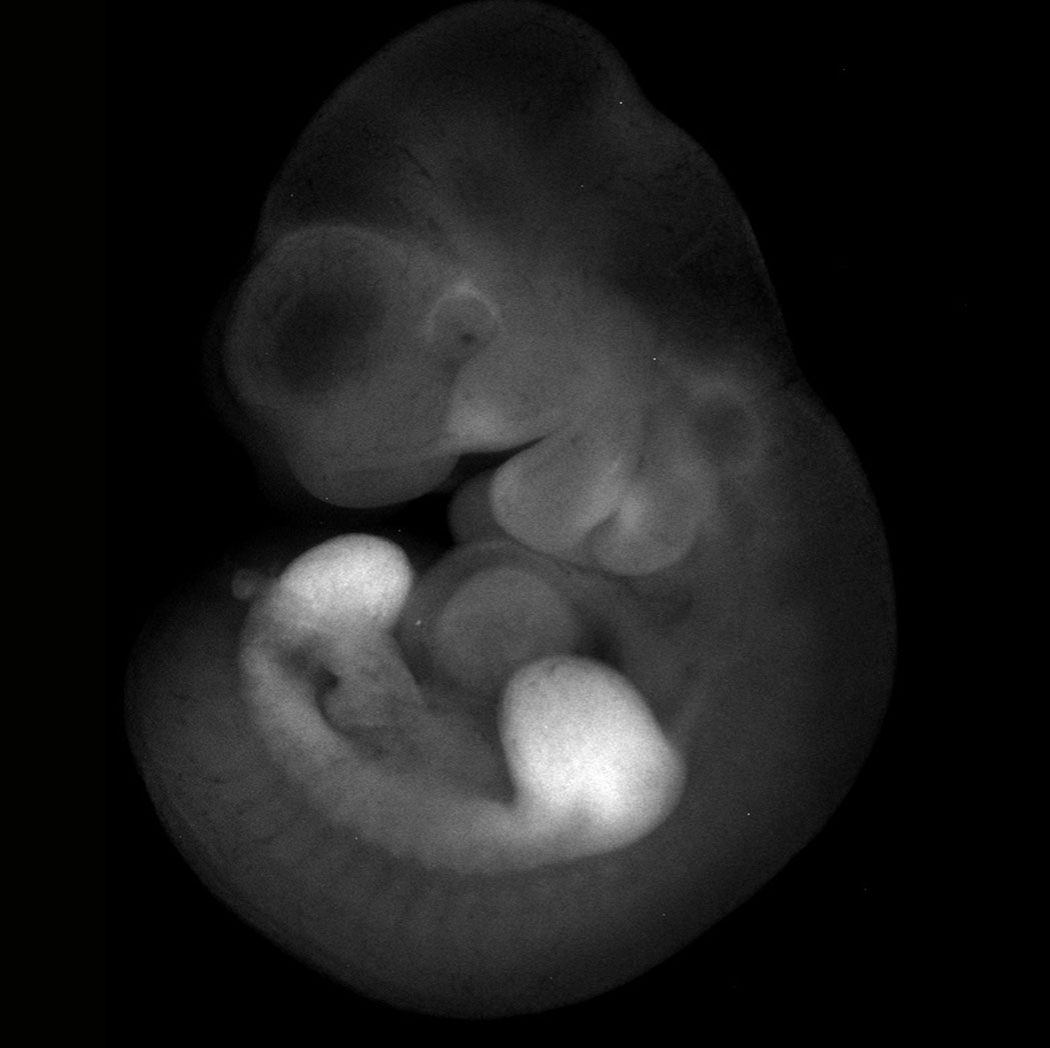
**A transgenic mouse embryo expressing the *Prrx1* enhancer transgene that highlights mesenchyme of the limb buds, flank and parts of the craniofacial mesenchyme.**

**What's next for you?**

I'm excited to start new projects in my lab! I think there is so much we can learn from comparing mechanisms of repair and regeneration across species.

**Were there other team members who were integral to this project?**

This was a joint project with Tatiana Sandoval-Guzmán, a group leader at the Center for Regenerative Therapies in Dresden. She is a wonderful collaborator and experimentalist, which made this project a lot of fun to work on. We also had some great help from Lidia Grosser, a graduate student in the Tanaka lab. She made many of the beautiful micrographs of embryonic limbs!
